# Lower Limit of Normal of Pulmonary Function to Define Baseline Lung Allograft Dysfunction

**DOI:** 10.1097/TXD.0000000000001913

**Published:** 2026-02-23

**Authors:** Michael Gerckens, Susanne Simon, Carlo Mümmler, Paola Arnold, Jürgen Barton, Ali Önder Yildirim, Jürgen Behr, Nikolaus Kneidinger, Jens Gottlieb

**Affiliations:** 1 Department of Medicine V, University Hospital, LMU Munich, Comprehensive Pneumology Center (CPC-M), German Center for Lung Research (DZL), Munich, Germany.; 2 Institute of Lung Health and Immunity (LHI), Helmholtz Munich, Comprehensive Pneumology Center (CPC-M)/Member of the German Center for Lung Research (DZL), Munich, Germany.; 3 Department of Respiratory Medicine and Infectious Diseases, Hannover Medical School, Hannover, Germany.; 4 Institute of Experimental Pneumology, LMU University Hospital, Ludwig Maximilians University of Munich, LMU Munich, Munich, Germany.; 5 Department of Internal Medicine, Division of Respiratory Medicine, Lung Research Cluster, Medical University of Graz, Graz, Austria.; 6 German Center for Lung Research (DZL), Hannover, Germany.

## Abstract

**Background.:**

Subnormal lung function after lung transplantation (LTx) has increasingly been recognized as an independent risk factor for mortality. Historically, baseline lung allograft dysfunction (BLAD) has been defined using the fixed “< 80% predicted” threshold from population-wide reference equations, which disregards age- and sex-related variability in spirometric values and can lead to systematic overdiagnosis, particularly in older and female recipients. While the lower limit of normal (LLN), derived from Global Lung Initiative reference equations, has been accepted as technical standard in spirometry, it has not yet been applied to define BLAD.

**Methods.:**

A retrospective multicenter study included LTx recipients transplanted between 2014 and 2018. Lung function trajectories and allograft survival were followed-up until August 2024. The association of BLAD defined by forced expiratory volume in 1 s (FEV1) or forced vital capacity (FVC) < LNN as time-dependent variable with graft loss was studied using time-dependent Cox proportional hazard models.

**Results.:**

We analyzed 726 patients after LTx including 102 unilateral LTx recipients, of whom 470 (65%) of the cohort achieved normal baseline lung function defined as FEV1 and FVC ≥ LLN. Two hundred thirty-six patients experienced graft loss (n = 2 redo LTx) and 179 developed chronic lung allograft dysfunction. After adjusting for age, disease, transplant type, and chronic lung allograft dysfunction, baseline FEV1 or FVC < LLN was associated with graft loss (hazard ratio, 1.822; 95% confidence interval, 1.372-2.418; *P* < 0.001).

**Conclusions.:**

BLAD defined by concurrent baseline FEV1 or FVC below the LLN was strongly associated with increased risk of graft loss. These findings extend prior studies that used the fixed 80% threshold by demonstrating that an LLN-based, age- and sex-adjusted definition of BLAD identifies lung transplant recipients at risk, thereby avoiding fixed cutoff associated age- and sex-bias.

## Graphical abstract

**Figure:**
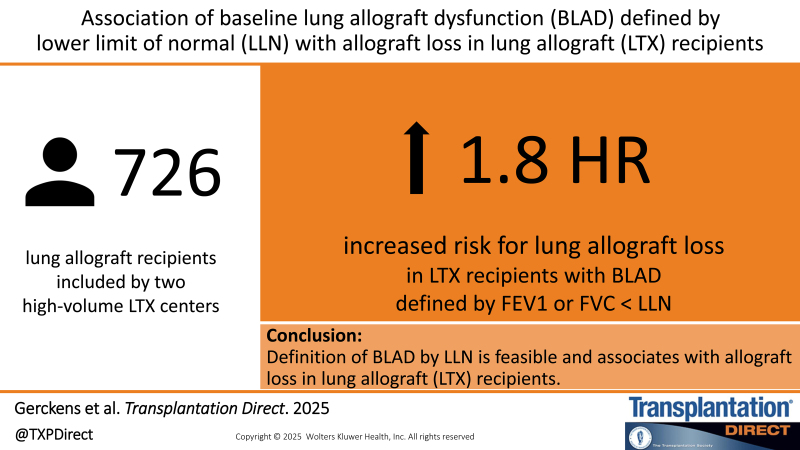

## INTRODUCTION

Monitoring allograft health after lung transplantation (LTx) encompasses a number of structural and functional assessments of the allograft, with serial spirometry measurements playing a pivotal role as a cost-efficient and noninvasive procedure offering high diagnostic value at a low cost.

While chronic lung allograft dysfunction (CLAD), defined as a significant decline in lung function compared with an individual baseline lung function, has been subject of intense investigation for the last decades,^1^ the failure to reach a normal baseline lung function, termed baseline lung allograft dysfunction (BLAD) is drawing increasing attention in the past years.^2-5^

Mohanka et al^6^ and Paraskeva et al^7^ demonstrated in their landmark analyses the association between abnormal spirometry values during the first year after transplantation and lung allograft recipients’ survival. Approaching baseline lung function as a dynamic factor that might improve over time has been investigated in several studies: Liu et al,^2^ Keller et al,^5^ Yamaguchi et al,^8^ as well as our group^4^ have shown associations between abnormal spirometry classified by fixed percentage predicted values and decreased survival.

In all of these studies, the thresholds for defining suboptimal baseline lung function were defined using percentage predicted values derived from lung function reference equations, such as the Global Lung Initiative (GLI),^9^ Crapo et al,^10^ and others. The frequently used threshold of 80% predicted from reference equations aligns with its historical role in distinguishing pathologically decreased lung function values from those that are still considered normal, leading to overdiagnosis of abnormal spirometric patterns, including airflow obstruction and restriction, particularly among older and female patients.^11^

The fixed 80% of predicted cutoff assumes that the distribution of lung function is the same across all ages and body sizes. Statistically, this is not correct, because spirometric values follow a normal distribution around the predicted mean with a certain SD. As people get older, the mean declines and the variation widens (heteroscedasticity). Using a fixed 80% cutoff ignores this variation and leads to systematic misclassification and a systematic overdiagnosis in older and female individuals.

The lower limit of normal (LLN) is defined as the value below the 5th percentile (approximately –1.645 SDs) of the reference population after adjusting for age, sex, height, and ethnicity. This statistical definition identifies the lowest 5% of values as abnormal, regardless of the absolute predicted value. In this way, LLN provides a more statistically valid threshold, ensuring that the classification of “normal” versus “abnormal” reflects the distribution of lung function in the population, rather than an arbitrary percentage and avoids sex- and age-biased overdiagnosis of disease.

Therefore, the use of the LLN has gained value over fixed percentage threshold in assessing lung health and is recognized as technical standard in interpretation of spirometry.^12^ LLN and *Z* scores are based on statistical norms and SDs from a reference population. This approach stands in contrast to the use of the fixed 80% predicted threshold not taking into account that the variability of the measured lung function value is not proportional to the predicted value.^11^ Thus, LLN provides a more individualized assessment of the patient’s lung function accounting for population specific bias and age-dependent dispersion. Thus, it enhances sensitivity and specificity of diagnosing respiratory conditions by focusing on statistical normality.^13^ Many guidelines have acknowledged the advantages of using LLN over fixed percentage threshold to avoid over- and underdiagnosis.^12,14^ However, all previous studies published on BLAD in double lung transplant recipients solely relied on the 80% predicted cutoff, despite those limitations.

The aim of this study was to investigate BLAD defined by the LLN defined by the GLI reference equations as risk factor for graft loss in lung allograft recipients using time-dependent Cox regression models.

## MATERIALS AND METHODS

A retrospective multicenter cohort study was performed in the 2 largest German lung transplant centers (Hannover and Munich) to assess baseline forced vital capacity (FVC) and forced expiratory volume in 1 s (FEV1) defined by the GLI reference equations compared with the respective LLN.

This study included all adult patients attending the specialized outpatient follow-up clinics and undergoing primary LTx between January 1, 2014, and December 31, 2018. Patients with a follow-up and survival of <1 y and patients with insufficient spirometric tests to calculate a baseline were excluded. Lung allograft survival was defined as time until death or retransplantation. Follow-up was recorded until August 31, 2024.

The study was performed according to the principles of the Declaration of Helsinki. Both centers are part of the German Center of Lung Research. The study was covered by the institutional ethics committee´s vote of institutional review board/ethics committee of the Hannover Medical School (No 2923-2015, update September 24, 2021) and by institutional ethics committee´s vote of institutional review board/ethics committee of the Ludwig-Maximilians-University of Munich (21-0020). Written consent was obtained from all patients included at the Hannover side. Institutional ethics committee´s vote of institutional review board of the Ludwig-Maximilians-University of Munich (21-0020) waived the need for informed consent for this retrospective study.

The primary outcome of this study was graft loss defined as death or retransplantation, whatever occurred first. Baseline FEV1 and FVC lung function parameters within the first year after LTx were defined as predictors for aforementioned outcomes.

Age at transplantation, transplant type, transplant indication (diagnosis), and obstruction in pulmonary function testing and CLAD were included as covariates into the multivariable analysis. Obstruction in pulmonary function testing and CLAD were modeled as time-dependent covariates. CLAD was defined and phenotyped according to the 2019 International Society for Heart & Lung Transplantation consensus CLAD definition.^1^ The baseline for BLAD designation used the 2 highest postoperative FEV1 values separated by a minimum of 3 wk, and the corresponding FVC values analogous to Verleden et al.^1^ Obstruction was defined as current FEV1/FVC ratio lower than the FEV1/FVC LLN calculated by the GLI reference equation. Manual correction of baseline FEV1, as used in the CLAD consensus report,^1^ was not applied.

BLAD was defined as baseline FEV1 or baseline FVC < LLN, as time-dependent variable. Furthermore, exploratory analyses associating baseline FEV1 and FVC < LLN as well as exclusively baseline FEV1 or baseline FVC < LLN were performed additionally. Time of reaching normal baseline lung allograft function was defined as the first of both lung function timepoints forming the baseline.

Translating the BLAD grading system from the % predicted definition of LLN-based BLAD definition, BLAD severity was graded according to the degree of deviation of FEV1 *Z* scores from the predicted mean, distinguishing mild (LLN to –2.5), moderate (–2.5 to –4), and severe (<–4) impairment, in accordance with the European Respiratory Society/American Thoracic Society technical standard.^12^

Baseline characteristics (eg, age, sex, underlying disease, lung transplant procedure: bilateral, unilateral, or combined organ transplantation) as well as lung function measurements were collected from electronic health records.

Reference values were based on recipients’ characteristics and on the 2022 GLI Global reference equations.^12^ Patient age at transplantation was used for calculation of the predicted FEV1, FVC, and FEV1/FVC ratio. Values below the LLN, derived from the GLI reference equations, of baseline FEV1, baseline FVC, and FEV1/FVC ratio were defined as abnormal.

### Statistics

Cohort demographics were reported with metric variables expressed as medians and 25% and 75% quartiles (interquartile range [IQR]) and categorical variables by absolute numbers and percentage of data entries. Interferential statistics was performed using the median test for continuous variables and the chi-square test or Fisher exact test for categorical variables.

Time-dependent Cox regression analysis was used to perform a univariate and multivariable analysis for evaluating the association between the dynamic evaluation of baseline FEV1, baseline FVC, and FEV1/FVC in relation to the respective LLN and lung allograft survival. Multivariable analysis also included CLAD diagnosis and obstruction in pulmonary function testing as dynamic risk factors and static variables such as transplant age, disease indication for transplantation, and transplantation type.

To compare the predictive performance of LLN versus 80% predicted-based BLAD definitions as previously described,^2^ we fitted multivariable Cox proportional hazards models using the counting-process formulation, with BLAD status as time-varying variable. BLAD status (LLN or 80% predicted) was included as a time-updated binary covariate with adjustment covariates matching the main models. Discrimination for death or retransplantation was assessed using Harrell’s C-index.

To assess the agreement between LLN-based and 80% fixed percentage based BLAD classifications over time, Cohen’s κ coefficients were calculated for each follow-up month after LTx. Confidence intervals (CIs) were calculated per patient-level nonparametric bootstrapping.

The level of significance was set at <0.05 for including variables identified by univariate analysis between groups. No imputation for missing data was performed.

## RESULTS

### Cohort Characterization

**FIGURE 1.**
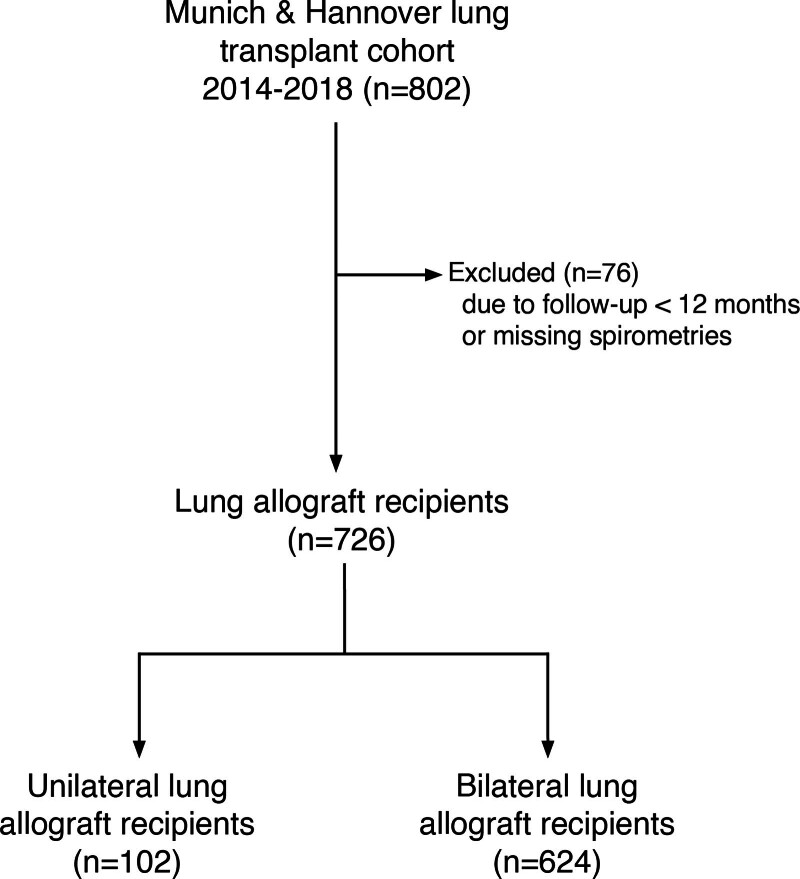
Study flow chart.

In total, 802 lung transplant recipients were screened for the study. Seven hundred twenty-six fulfilled inclusion criteria and were subsequently analyzed (Figure 1). Patient demographics are displayed in Table 1. The majority (624, 85%) were bilateral lung transplant recipients, 13% (102) underwent single LTx. Major exclusion criterion was graft loss after <12 mo, with 47 deaths and 2 retransplantations. Median follow-up of all included patients was 6.4 y (IQR, 5.0–7.9 y).

**TABLE 1.** Patient demographics

Parameters	Cohort (n = 726)
Age at transplant, y, median (25%–75% percentiles)	54 (43–60)
Donor age, y, median (25%–75% percentiles)	50 (36–60)
Donor smoking, n (%)	310 (43)
Sex, n (%)	
Female	346 (48)
Male	380 (52)
Type of transplant, n (%)	
Unilateral LTx	95 (13)
Bilateral LTx	618 (85)
Combined LTx	6 (4)
Lobar LTx	7 (1)
Ethnicity, n (%)	
Caucasian	718 (99)
African	1 (0.1)
Asian	3 (0.4)
Other/ mixed	4 (0.6)
Diagnosis, n (%)	
Emphysema/alpha-1 antitrypsin deficiency	201 (28)
Fibrosis/interstitial lung disease	306 (42)
Cystic fibrosis/bronchiectasis	132 (18)
Pulmonary hypertension/vascular diseases	39 (5)
Other	48 (7)
CLAD diagnosis, n (%)	179 (25)
CLAD type BOS	106 (15)
CLAD type RAS/mixed	34 (5)
CLAD of other type	39 (5)
Baseline lung function at end of follow-up, n (%)	
Baseline FEV1 and FVC > LLN	470 (65)
Baseline FEV1 and FVC < LLN	146 (20)
Baseline FEV1 > LLN only	77 (10)
Baseline FVC > LLN only	33 (5)

BOS, bronchiolitis obliterans syndrome; CLAD, chronic lung allograft dysfunction; FEV1, forced expiratory volume in 1 s; FVC, forced vital capacity; LLN, lower limit of normal; LTx, lung transplantation; RAS, restrictive allograft syndrome.

### Predicted Percentage Threshold Compared With LLN

Comparing FEV1 percentage predicted across age and sex of the investigated population revealed that LLN corresponded consistently to 79% predicted or smaller. Comparison across age groups and sex showed that the fixed 80% predicted cutoff and the LLN diverged progressively with increasing age, beginning around 30 y old. This divergence reflects the wider dispersion of normal FEV1 values at older ages. Differences between sexes were also observed, with lower % predicted values at the LLN in females compared with males (**Figure S1**, **SDC**, https://links.lww.com/TXD/A831). This illustrates that the LLN provides an age- and sex-adjusted threshold, thereby avoiding the systematic bias inherent to the fixed 80% cutoff.

### Baseline Lung Allograft Function

Four hundred seventy LTx recipients (65%) achieved a combination of baseline FEV1 > LNN and baseline FVC > LLN. Seventy-seven LTx recipients (11%) achieved a baseline FEV1 > LLN only, whereas 33 LTx recipients (5%) reached only baseline FVC > LNN. One hundred forty-six LTx recipients (20%) reached neither baseline FEV1 > LNN nor baseline FVC > LLN.

**FIGURE 2.**
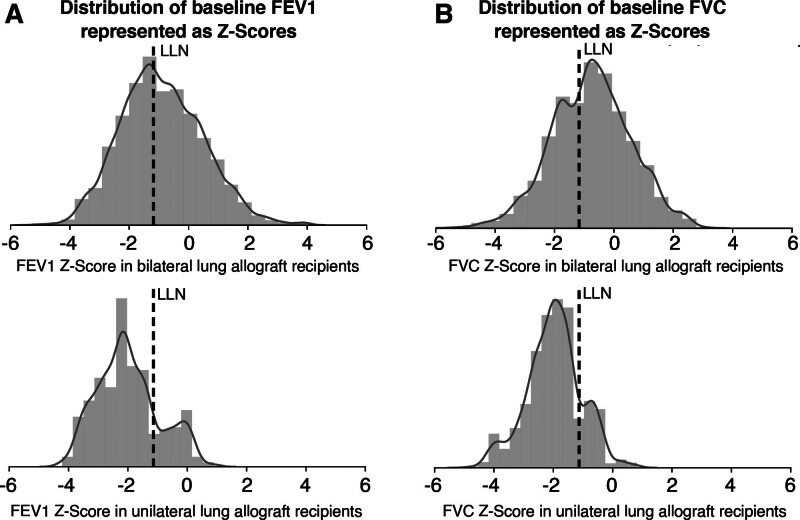
Distributions of baseline FEV1 and FVC represented as *Z* scores in bilateral and unilateral lung allograft recipients. FEV1, forced expiratory volume per 1 s; FVC, forced vital capacity; LLN, lower limit of normal.

Baseline FEV1 > LLN after LTx was reached by 464 of 624 (74%) bilateral LTx recipients (including combined transplantations) at a median of 54 d after LTx (IQR, 33–103 d). Thirty-seven of 102 (36%) size reduced LTx recipients (unilateral and lobar transplantations) reached a baseline FEV1 > LLN at a median of 68 d after LTx (IQR, 52–112 d). In bilateral LTx, recipients reached a median baseline of 85% predicted (*Z* score –1.01) and 86% predicted (*Z* score –0.96) for FEV1 and FVC, respectively. In unilateral LTX, recipients achieved a median baseline of 64% predicted (*Z* score –2.11) and 67% predicted (*Z* score –2.09) for FEV1 and FVC, respectively (Figure 2).

Agreement analyses between the LLN-based and 80% predicted-based BLAD classifications demonstrated only moderate overall agreement, indicating that a relevant subset of patients was reclassified when applying the LLN-based approach: Agreement decreased with increasing age and was consistently lower among females compared with males (**Figure S2**, **SDC**, https://links.lww.com/TXD/A831).

### Outcomes

Two hundred thirty-six patients died (n = 234) or underwent retransplantation (n = 2) during the study period.

Exploratory univariate time-dependent Cox regression for lung allograft survival analyses looking only at FEV1 or FVC alone showed that baseline FEV1 < LLN was associated with a significantly decreased allograft survival (hazard ratio [HR], 1.936; 95% CI, 1.492-2.511), similarly for baseline FVC (1.967; 95% CI, 1.514-2.556; Table 2).

**TABLE 2.** Time-dependent Cox regression allograft survival analysis for (I) baseline FEV1 < LLN compared with reference (baseline FEV1 ≥ LLN), (II) baseline FVC < LLN compared with reference (baseline FVC ≥ LLN), and for (III) baseline FVC and/or FEV1 < LLN compared with reference (both baseline FEV1 and FVC ≥ LLN)

Variables	Category	Hazard ratio (95% confidence interval)	*P*
Time-dependent Cox regression analysis I			
Baseline FEV1	FEV1 ≥ LLN	Reference	
	FEV1 < LLN	1.936 (1.492-2.511)	<0.001
Time-dependent Cox regression analysis II			
Baseline FVC	FVC ≥ LLN	Reference	
	FVC < LLN	1.967 (1.514-2.556)	<0.001
Time-dependent Cox regression analysis III			
Baseline FEV1 and FVC	FEV1 and FVC ≥ LLN	Reference	
	FEV1 < LLN only	1.278 (0.790-2.054)	0.31
	FVC < LLN only	1.272 (0.762-2.144)	0.37
	FEV1 and FVC < LLN	2.313 (1.737-3.079)	<0.001

FEV1, forced expiratory volume in 1 s; FVC, forced vital capacity; LLN, lower limit of normal.

When categorizing in (a) both baseline FEV1 and FVC < LLN (defining BLAD), or (b) only baseline FEV1 < LLN, or (c) baseline FVC only < LLN, time-dependent Cox regression analysis showed a significant increase in mortality for a combination of baseline FEV1 and FVC < LLN (HR, 2.313; 95% CI, 1.737- 3.079) but not for baseline FEV1 < LLN only or FVC < LLN only (Table 2).

One hundred seventy-nine patients (25%) developed CLAD with bronchiolitis obliterans syndrome as the predominant phenotype (59%), 34 (19%) had restrictive allograft syndrome (RAS) or mixed phenotype. Sixty-three patients (27%) died from CLAD as documented cause of death.

Univariate Cox regression analysis found unilateral LTx to be associated with shorter lung allograft survival. Further, increased age at LTx was associated with shorter allograft survival. Regarding underlying transplant indication, cystic fibrosis/bronchiectasis disease was associated with a longer allograft survival. Regarding pulmonary function testing, obstruction, defined as spirometric FEV1/FVC < LNN, was identified as time-dependent risk factor for shorter allograft survival (HR, 1.916; 95% CI, 1.479-2.483). All CLAD phenotypes were associated with shorter survival, most pronounced for RAS (Table 3).

**TABLE 3.** Lung allograft survival analysis by univariate and multivariable time-dependent Cox regression analysis

Variable	Category	n	Univariable Cox regression analysis		Multivariable Cox regression analysis	
			Hazard ratio (95% confidence interval)	*P*	Hazard ratio (95% confidence interval)	*P*
Transplant type	Bilateral (incl. combined)	624	Reference		Reference	
	Unilateral (incl. lobar)	102	2.105 (1.060-2.886)	<0.001	1.002 (0.690-1.456)	0.990
BLAD^*a*^	Baseline FEV1 and FVC ≥ LLN	^*b*^	Reference		Reference	
	Baseline FEV1 or FVC < LLN (BLAD)	^*b*^	1.842 (1.424-2.382)	<0.001	1.822 (1.372-2.418)	<0.001
Obstruction^*a*^	FEV1/FVC ≥ LLN	^*b*^	Reference		Reference	
	FEV1/FVC < LLN	^*b*^	1.916 (1.479-2.483)	<0.001	1.486 (1.093-2.020)	0.011
Diagnosis	COPD/emphysema	201	Reference		Reference	
	Pulmonary vascular diseases	39	0.931 (0.515-1.674)	0.811	1.393 (0.709-2.738)	0.336
	Cystic fibrosis/bronchiectasis	132	0.478 (0.309-0.741)	<0.001	0.843 (0.476-1.495)	0.560
	Pulmonary fibrosis	306	0.859 (0.641-1.150)	0.306	0.910 (0.670-1.236)	0.546
	Other	48	0.621 (0.338-1.139)	0.124	0.658 (0.348-1.242)	0.197
Age category	38–50 y	155	Reference		Reference	
	<38 y	144	1.253 (0.779-2.016)	0.353	0.222 (0.716-2.178)	0.433
	51–57 y	165	1.681 (1.090-2.592)	0.019	1.811 (1.161-2.824)	0.009
	58–61 y	115	1.935 (1.225-3.053)	0.004	2.014 (1.257-3.227)	0.004
	>61 y	147	2.947 (1.949-4.454)	<0.001	2.949 (1.865-4.661)	<0.001
CLAD^*a*^	No CLAD	^*b*^	Reference		Reference	
	BOS phenotype	^*b*^	3.791 (2.697-5.328)	<0.001	2.986 (2.042-4.368)	<0.001
	RAS/mixed phenotype	^*b*^	5.000 (3.379-7.400)	<0.001	4.109 (2.726-6.195)	<0.001
	Phenotype, other/unknown	^*b*^	3.941 (1.595-9.734)	<0.001	2.829 (1.106-7.237)	0.030

^*a*^Modeled as time-dependent covariate.

^*b*^Modeled as time-dependent covariate; category membership may change over time; hence, baseline absolute counts (n) are not applicable.

BLAD, baseline lung allograft dysfunction; BOS, bronchiolitis obliterans syndrome; CLAD, chronic lung allograft dysfunction; COPD, chronic obstructive pulmonary disease; FEV1, forced expiratory volume in 1 s; FVC, forced vital capacity; LLN, lower limit of normal; RAS, restrictive allograft syndrome.

Multivariable time-dependent Cox regression analysis of the aforementioned covariates demonstrated concurrent baseline FEV1 or FVC < LLN was significantly associated with shorter allograft survival (Table 3; **Figure S3**, **SDC**, https://links.lww.com/TXD/A831). The multivariable model was designed to evaluate the association between BLAD and graft loss. Coefficients of adjustment covariates are shown for transparency only and should not be interpreted as independent effects.^15^

Compared with the main analysis presented in Table 3, defining BLAD as FEV1 or FVC < LLN. **Table S1** (**SDC**, https://links.lww.com/TXD/A831) provides a complementary view by also assessing isolated impairments of FEV1 or FVC. Here, the combined impairment of FEV1 and FVC < LLN was significantly associated with reduced allograft survival, while isolated impairments were not.

In an exploratory analysis, we compared the discriminatory ability of LLN- and 80% predicted-based BLAD definitions using multivariable Cox models with time-updated BLAD status. Discrimination was very similar for both approaches with a Harrell’s C-index of 0.724 (95% CI, 691-0.757) and 0.716 (95% CI, 0.681-0.751) for the LLN-based definition and for the 80% predicted definition, respectively.

In a further exploratory analysis, CLAD was not more likely in BLAD patients, with a time-dependent Cox regression analysis regarding time to CLAD, not providing evidence that LTx recipients with BLAD have a higher or lower risk of developing CLAD (HR for baseline FEV1 or FVC < LLN, 1.185; 95% CI, 0.861-1.629). Moreover, increasing BLAD severity defined by LLN was associated with progressively shorter graft survival (**Table S2**, **SDC**, https://links.lww.com/TXD/A831).

## DISCUSSION

To our knowledge, this is the first study using LLN to define BLAD after LTx. To date, all studies investigating BLAD have relied on a fixed 80% predicted cutoff. As a result, a fixed percentage threshold leads to systematic overdiagnosis in elderly and female patients. The LLN, defined as the 5th percentile of the reference population adjusted for age, sex, height, and ethnicity, avoids these limitations and provides a statistically valid, individualized threshold. Concordance agreement between the LLN-based and 80% predicted-based BLAD classifications decreased with age and was consistently lower among females compared with males (**Figure S2**, **SDC**, https://links.lww.com/TXD/A831), supporting the notion that reliance on fixed % predicted thresholds may lead to systematic overdiagnosis of BLAD in elderly and female patients.

Our study is the first to demonstrate that BLAD, also when defined by LLN, is strongly associated with graft loss, introducing a definition that aligns with current standards in pulmonary function testing. This approach may provide the lung transplant community with a more precise and unbiased framework for identifying patients at risk.

Defining thresholds for normal versus abnormal baseline lung function after LTx is an ongoing matter of debate. Several publications used an 80% predicted threshold for baseline FEV1 and baseline FVC; however, these were based on different reference equations (Liu et al,^2^ Li et al,^3^ and Keller et al^5^). Recently, published studies have identified the different definitions as a source of variability in the presented associations.^16^ While the continued use of definitions based on percentage predicted threshold will ensure better comparability with earlier studies (disregarding differences in previous definitions), one has to acknowledge that international technical guides to pulmonary function interpretation^13,17^ as well as studies^11,18-20^ and guidelines^21^ in other respiratory condition areas^22^ increasingly prefer *Z* score and LLN over the use of percentage predicted. The comparison of LLN values stratified by sex and age with fixed percentage predicted thresholds, as shown in **Figure S1** (**SDC**, https://links.lww.com/TXD/A831), highlights a striking discrepancy: the fixed 80% predicted cutoff substantially overestimates the prevalence of suboptimal lung function, particularly among older individuals and even more markedly in women. This illustrates a key limitation of using fixed cutoffs ignoring age- and sex-dependent heteroscedasticity inherent in lung function. As a result, reliance on the 80% threshold risks systematic overdiagnosis of BLAD in elderly and female patients. This underscores the importance of using LLN-based thresholds, which are now widely recommended in pulmonary function testing for their ability to account for natural population variability.

Beyond the time-varying HRs, our exploratory analyses comparing LLN- and 80% predicted-based BLAD definitions showed nearly identical predictive performance. Using Cox models with time-updated BLAD status, Harrell’s C-indices for subsequent death or retransplantation were very similar with overlapping CIs, indicating no clinically meaningful difference in discrimination within the limits of our sample size and event numbers. These findings align with previous work in other lung diseases,^19,23,24^ where LLN- and fixed-threshold definitions typically yield comparable prognostic discrimination despite differences in individual classification. In our cohort, this suggests that the choice between LLN and an 80% predicted cutoff does not materially alter survival prediction, supporting the prognostic validity of earlier studies that used fixed thresholds.

At the same time, these results do not imply that LLN and 80% predicted are interchangeable concepts. LLN retains clear methodological advantages, most notably its physiologic grounding and avoidance of systematic misclassification across age and body size strata, as demonstrated in our main analyses.

The rationale for adopting LLN lies in statistical validity: spirometric values follow a normal distribution with increasing dispersion at older ages (heteroscedasticity), which the fixed 80% cutoff does not take into account. In contrast, LLN defines abnormality as values below the 5th percentile of a reference population adjusted for age, sex, height, and ethnicity. By applying this individualized threshold, our study demonstrates that BLAD defined by LLN is strongly associated with graft loss, extending prior work based on the fixed 80% cutoff and aligning the definition of BLAD with current standards in pulmonary function testing.

Our results show that applying the LLN as threshold to define BLAD is feasible. BLAD defined by baseline FEV1 or FVC < LLN is associated with significantly shorter allograft survival (Table 2). Effects on allograft survival are weaker if only baseline FEV1 or even only baseline FVC is below LLN (Table 2). Multivariable time-dependent Cox regression analysis highlights baseline FEV1 and FVC < LLN as independent risk factor, with spirometric obstruction, increasing age, and CLAD bronchiolitis obliterans syndrome phenotype and CLAD RAS phenotype as confounders (Table 3).

No association between BLAD prevalence and CLAD incidence were seen, which is in line with previous BLAD studies and indicates that CLAD and BLAD are separate entities with different underlying pathomechanisms.

Exploratory analysis analyzing isolated FEV1 or FVC impairments separately (**Table S1**, **SDC**, https://links.lww.com/TXD/A831) highlights that patients with isolated FEV1 or FVC impairment did not show significantly worse outcomes than patients without BLAD. Nevertheless, we used a BLAD definition of FEV1 or FVC < LLN to maintain comparability with previous study and upcoming consensus guidelines.

LLN corresponds to systematically lower percentage predicted, varying by sex and age of the lung transplant recipient, but generally lower than the fixed 80% cutoff. Consequently, patients reach baseline lung allograft function earlier after transplantation compared with previous BLAD definitions. We demonstrate BLAD defined by LLN as a risk factor for limited allograft survival with a similar effect size to previous publications and characterize BLAD as independent risk factor from CLAD and other known risk factors. We also propose BLAD grades defined by LLN: Grading BLAD severity based on *Z* scores provides additional granularity and supports the prognostic relevance of the LLN-based framework, as greater deviations from the predicted mean were associated with progressively worse outcomes. We aimed to provide a grading approach analogous to the % predicted BLAD definition presented earlier, ensuring comparability across frameworks.

In summary, this study provides arguments for the use of LLN and against the use of fixed 80% percentage predicted cutoffs, as widely accepted for the diagnosis by pulmonary testing. A multitude of other pulmonary disease areas have adapted LLN for diagnosis successfully, such as in COPD,^25^ cystic fibrosis,^22^ or asthma.^26^

Overall, the appropriateness of criteria and thresholds for defining BLAD is somewhat open for interpretation. Criteria that more distinctly differentiate between patients with high and low mortality risk might be preferred for BLAD definition. However, only anchoring the BLAD definition to mortality might hold caveats, as the association between BLAD and mortality risk may be influenced by underlying (latent) clinical factors, not directly linked to BLAD.

We believe our study holds several strengths, including a multicenter design with a large number of patients for the shown comprehensive (multivariable) analyses including a wide spectrum of transplant indications, as well as a wide range of transplant age. We treated pulmonary function testing results as well as CLAD diagnosis as time-dependent covariates acknowledging the dynamic nature of those risk factors.

Despite being, to our knowledge, the largest study on BLAD by patient number, a limitation of this study is center- and country-specific effects still exist and may limit generalizability of the results, that would ideally be replicated in a study with more centers, also addressing the limited ethnic diversity in the study cohort. With respect to potential confounders included in the analysis, residual confounding from prior episodes of acute rejection or infection, data not available in this study, cannot be excluded. Also, removing lung allograft recipients with short survival precludes any conclusions on associations between BLAD and early mortality.

To date, BLAD remains a spirometric phenomenon, most likely caused by a diverse set of etiologies. However, a precise definition could aid the elucidation of pulmonary and extrapulmonary causes.^16^ Epidemiological studies showed that incorporating sex and age-specific normal range into the spirometry interpretation by using LLN avoids under- or overdiagnosis in specific sets of patients, which might hold true in lung allograft recipients as well.

## Supplementary Material



## References

[1] Verleden GM Glanville AR Lease ED. Chronic lung allograft dysfunction: definition, diagnostic criteria, and approaches to treatment—a consensus report from the Pulmonary Council of the ISHLT. J Heart Lung Transplant. 2019;38:493–503. 30962148 10.1016/j.healun.2019.03.009

[2] Liu J Jackson K Weinkauf J. Baseline lung allograft dysfunction is associated with impaired survival after double-lung transplantation. J Heart Lung Transplant. 2018;37:895–902. 29602706 10.1016/j.healun.2018.02.014

[3] Li D Weinkauf J Kapasi A. Baseline lung allograft dysfunction in primary graft dysfunction survivors after lung transplantation. Respir Med. 2021;188:106617. 34571454 10.1016/j.rmed.2021.106617

[4] Gerckens M Mummler C Richard A. Characterization of baseline lung allograft dysfunction in single lung transplant recipients. Transplantation. 2024;109:e213–e221. 39250332 10.1097/TP.0000000000005189

[5] Keller MB Sun J Alnababteh M. Baseline lung allograft dysfunction after bilateral lung transplantation is associated with an increased risk of death: results from a multicenter cohort study. Transplant Direct. 2024;10:e1669. 38953039 10.1097/TXD.0000000000001669 PMC11216668

[6] Mohanka MR Kanade R Garcia H. Significance of best spirometry in the first year after bilateral lung transplantation: association with 3-year outcomes. Transplantation. 2020;104:1712–1719. 32732851 10.1097/TP.0000000000003046 PMC7373484

[7] Paraskeva MA Borg BM Paul E. Abnormal one-year post-lung transplant spirometry is a significant predictor of increased mortality and chronic lung allograft dysfunction. J Heart Lung Transplant. 2021;40:1649–1657. 34548197 10.1016/j.healun.2021.08.003

[8] Yamaguchi M Kawashima M Muraoka T. Baseline lung allograft dysfunction after bilateral deceased-donor lung transplantation: a single-center experience in Japan. Respir Investig. 2024;62:838–843. 10.1016/j.resinv.2024.07.009 39047315

[9] Quanjer PH Stanojevic S Cole TJ; ERS Global Lung Function Initiative. Multi-ethnic reference values for spirometry for the 3-95-yr age range: the global lung function 2012 equations. Eur Respir J. 2012;40:1324–1343. 22743675 10.1183/09031936.00080312 PMC3786581

[10] Crapo RO Morris AH Gardner RM. Reference spirometric values using techniques and equipment that meet ATS recommendations. Am Rev Respir Dis. 1981;123:659–664. 7271065 10.1164/arrd.1981.123.6.659

[11] Miller MR Quanjer PH Swanney MP. Interpreting lung function data using 80% predicted and fixed thresholds misclassifies more than 20% of patients. Chest. 2011;139:52–59. 20522571 10.1378/chest.10-0189

[12] Stanojevic S Kaminsky DA Miller MR. ERS/ATS technical standard on interpretive strategies for routine lung function tests. Eur Respir J. 2022;60:2101499. 34949706 10.1183/13993003.01499-2021

[13] Stanojevic S Wade A Stocks J. Reference values for lung function: past, present and future. Eur Respir J. 2010;36:12–19. 20595163 10.1183/09031936.00143209

[14] Quanjer PH Brazzale DJ Boros PW. Implications of adopting the global lungs initiative 2012 all-age reference equations for spirometry. Eur Respir J. 2013;42:1046–1054. 23520323 10.1183/09031936.00195512

[15] Lederer DJ Bell SC Branson RD. Control of confounding and reporting of results in causal inference studies. Guidance for authors from editors of respiratory, sleep, and critical care journals. Ann Am Thorac Soc. 2019;16:22–28. 30230362 10.1513/AnnalsATS.201808-564PS

[16] Rullay A Kaur K Holman J. Health-related quality of life and exercise capacity in double lung transplant recipients with baseline lung allograft dysfunction. Transplant Direct. 2025;11:e1751. 39802201 10.1097/TXD.0000000000001751 PMC11723686

[17] Ong-Salvador R Laveneziana P de Jongh F. ERS/ATS Global Lung Function initiative normal values and classifying severity based on z-scores instead of per cent predicted. Breathe (Sheff). 2024;20:230227. 39660084 10.1183/20734735.0227-2023 PMC11629165

[18] Cestelli L Johannessen A Gulsvik A. Risk factors, morbidity, and mortality in association with preserved ratio impaired spirometry and restrictive spirometric pattern: clinical relevance of preserved ratio impaired spirometry and restrictive spirometric pattern. Chest. 2024;167:548–560. 39209063 10.1016/j.chest.2024.08.026

[19] Mannino DM Diaz-Guzman E. Interpreting lung function data using 80% predicted and fixed thresholds identifies patients at increased risk of mortality. Chest. 2012;141:73–80. 21659434 10.1378/chest.11-0797

[20] Kanj AN Niven AS. Race-neutral z-score classification of airflow obstruction: a measured step forward. Am J Respir Crit Care Med. 2024;210:1287–1289. 38820133 10.1164/rccm.202404-0873ED PMC11622434

[21] Venkatesan P. GOLD COPD report: 2024 update. Lancet Respir Med. 2024;12:15–16. 38061380 10.1016/S2213-2600(23)00461-7

[22] Stanojevic S Stocks J Bountziouka V. The impact of switching to the new global lung function initiative equations on spirometry results in the UK CF registry. J Cyst Fibros. 2014;13:319–327. 24332996 10.1016/j.jcf.2013.11.006

[23] Colak Y Afzal S Nordestgaard BG. Prevalence, characteristics, and prognosis of early chronic obstructive pulmonary disease. The Copenhagen general population study. Am J Respir Crit Care Med. 2020;201:671–680. 31770495 10.1164/rccm.201908-1644OC PMC7068820

[24] Bhatt SP Balte PP Schwartz JE. Discriminative accuracy of FEV1:FVC thresholds for COPD-related hospitalization and mortality. JAMA. 2019;321:2438–2447. 31237643 10.1001/jama.2019.7233 PMC6593636

[25] Quanjer PH. Correctly defining criteria for diagnosing chronic obstructive pulmonary disease matters. Am J Respir Crit Care Med. 2014;189:230–230. 10.1164/rccm.201306-1031LE 24428654

[26] Quanjer PH Weiner DJ. Interpretative consequences of adopting the global lungs 2012 reference equations for spirometry for children and adolescents. Pediatr Pulmonol. 2014;49:118–125. 24115510 10.1002/ppul.22876

