# Supplementary material for: Lower Limit of Normal of Pulmonary Function to Define Baseline Lung Allograft Dysfunction
Source: Transplant Direct. 2026 Feb 23;12(3):e1913. doi: 10.1097/TXD.0000000000001913 (PMC12931945; doi:10.1097/TXD.0000000000001913)
Supplement: Supplementary file 1 [file txd-12-e1913-s001.pdf]

Supplemental figure 1

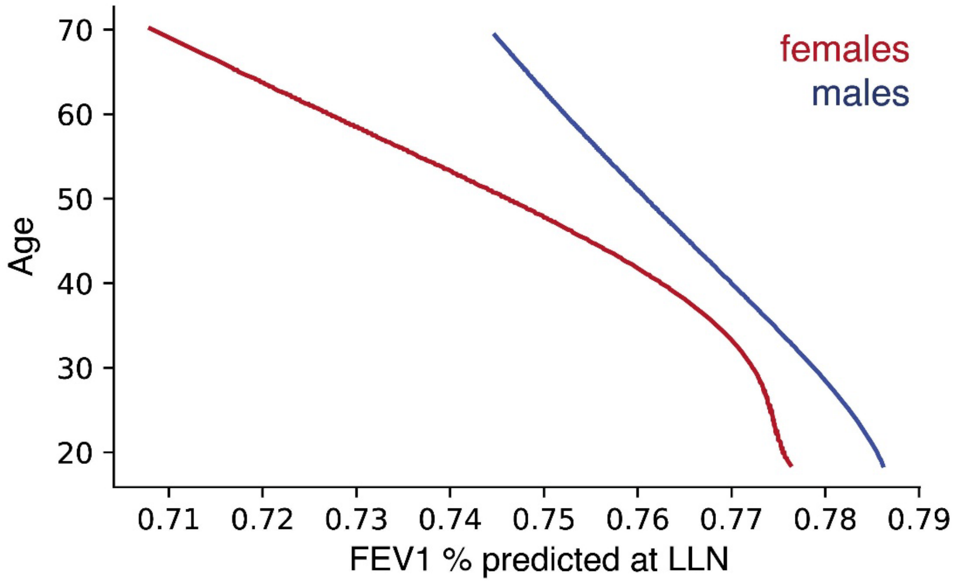

Supplemental figure 1: Relationship of FEV1 % predicted at LLN for female and male individuals as function of age.

Footnotes abbreviations: LLN lower limit of normal, FEV1 forced exspiratory volume per 1 second

Supplemental figure 2

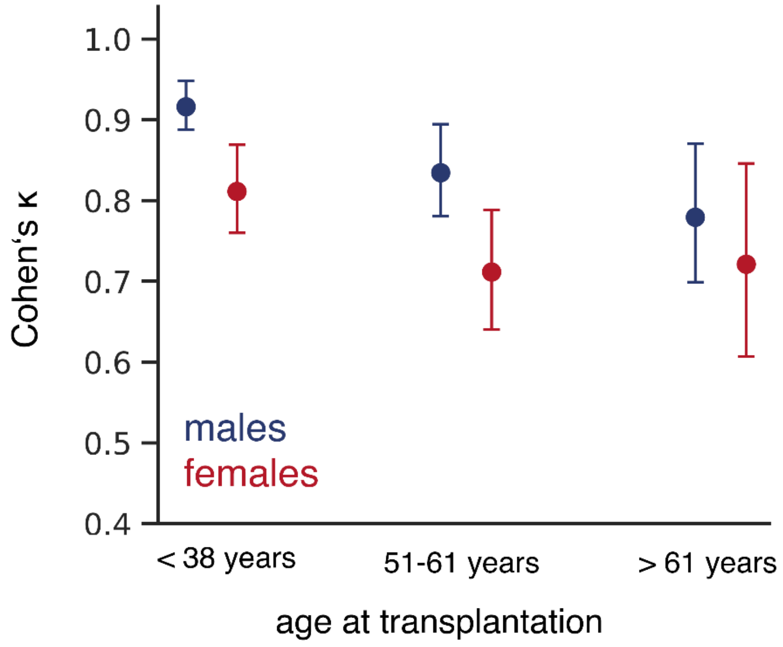

Figure S2: Agreement between LLN- and 80%-predicted–based classifications by age and sex. Cohen's κ coefficients (mean with 95 % confidence interval) for agreement between the LLN- and 80%-predicted–based classification approaches are shown, stratified by sex (red = females, blue = males) and age at transplantation. Agreement tended to decrease with increasing age in both sexes, with consistently lower κ values observed among females.

Supplemental figure 3

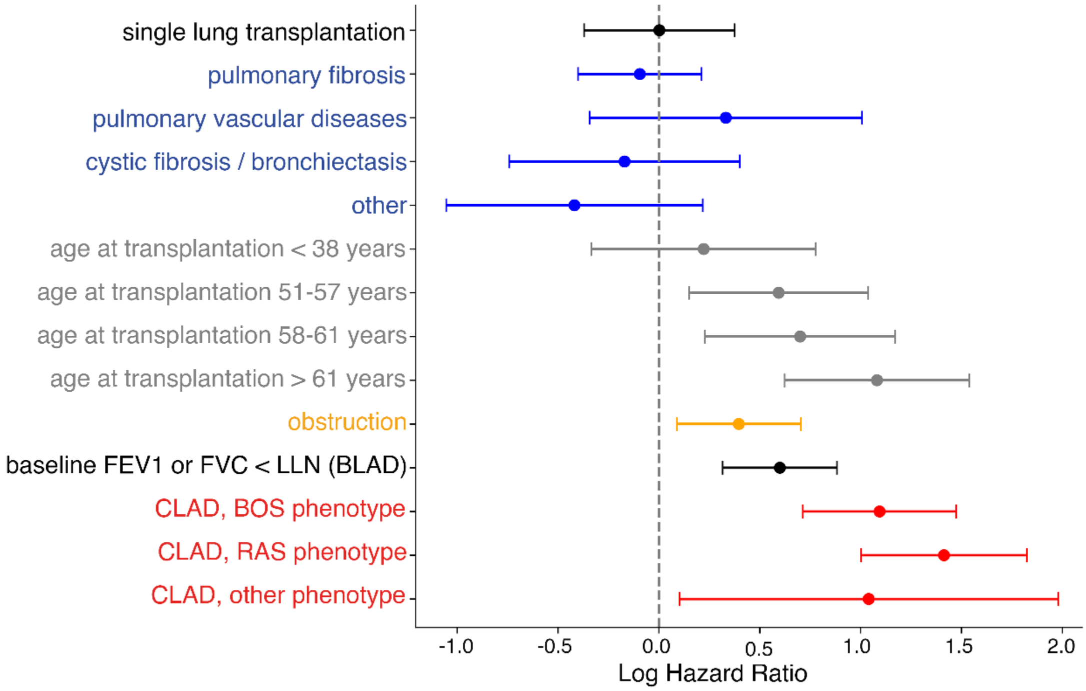

Supplemental figure 3: Forest plot visualizing multivariate proportional hazard cox regression model of factors associated with lung allograft loss (compare to Table 3).

Footnotes abbreviations: LLN lower limit of normal, FEV1 forced exspiratory volume per 1 second, FVC forced vital capacity, CLAD chronic lung allograft dysfunction, BOS bronchiolitis obliterans syndrome, RAS restrictive lung allograft syndrome

Table S1: Lung allograft survival analysis by univariate and multivariable time-dependent cox regression analysis, including separate assessment of isolated FEV1 or FVC impairment (FEV1 < LLN only, FVC < LLN only) and combined impairment (FEV1 and FVC < LLN; BLAD).

			Univariable Cox Regression Analysis			Multivariable Cox Regression Analysis		
variable	category	n	hazard ratio	95% confidence interval	p-value	hazard ratio	95% confidence interval	p-value
transplant	Bilateral (incl. combined)	624	reference			reference		
	**Unilateral (incl. lobar)**	**102**	**2.349**	**1.703-3.240**	**<0.001**	0.897	0.615-1.308	0.571
BLAD*[1]	Baseline FEV1 and FVC ≥ LLN	*[2]	reference			reference		
	Baseline FEV1 < LLN only	*[2]	1.278	0.790-2.054	0.31	1.328	0.799-2.207	0.273
	Baseline FVC < LLN only	*[2]	1.272	0.762-2.144	0.37	1.232	0.727-2.087	0.775
	**Baseline FEV1 and FVC < LLN**	***[2]**	**2.313**	**1.737-3.079**	**<0.001**	**2.406**	**1.737-3.331**	**<0.001**
Obstruction*[1]	FEV1/FVC ≥ LLN	*[2]	reference			reference		
	FEV1/FVC < LLN	*[2]	**1.916**	**1.479-2.483**	**<0.001**	**1.491**	**1.088-2.044**	**0.013**
Diagnosis	COPD/emphysema	201	reference			reference		
	Pulmonary vascular diseases	39	0.931	0.518-1.674	0.810	1.388	0.703-2.738	0.345
	**Cystic fibrosis / bronchiectasis**	**132**	**0.483**	**0.312-0.747**	**<0.001**	0.882	0.495-1.573	0.672
	Pulmonary fibrosis	306	0.859	0.641-1.149	0.306	0.901	0.662-1.308	0.510
	other	48	0.621	0.338-1.139	0.124	0.882	0.349-1.250	0. 574
Age	38 - 50 years	155	reference			reference		
	< 38 years	144	1.252	0.795-2.091	0.352	1.228	0.698-2.159	0.476
	**51 - 57 years**	**165**	**1.681**	**1.072-2.592**	**0.018**	**1.946**	**1.244-3.044**	**0.004**
	**58 – 61 years**	**115**	**1.935**	**1.226-3.054**	**0.005**	**2.149**	**1.337-3.453**	**0.002**
	**> 61 years**	**147**	**2.950**	**1.949-4.454**	**<0.001**	**3.081**	**1.952-4.863**	**<0.001**
CLAD*[1]	No CLAD	*[2]	reference			reference		
	**BOS phenotype**	*[2]	**3.791**	**2.697-5.328**	**<0.001**	**3.057**	**2.076-4.500**	**<0.001**
	**RAS/ mixed phenotype**	*[2]	**5.000**	**3.379-7.400**	**<0.001**	**4.183**	**2.783-6.287**	**<0.001**
	**phenotype, other/ unknown**	*[2]	**3.941**	**1.595-9.734**	**0.003**	2.629	1.023-6.758	0.045

*[1] modelled as time-dependent covariate, *[2] modelled as time-dependent covariate; category membership may change over time, hence baseline absolute counts (n) are not applicable.

Footnotes abbreviations: BLAD: baseline lung allograft dysfunction, CLAD: chronic lung allograft dysfunction, BOS: bronchiolitis obliterans syndrome, RAS: restrictive allograft syndrome, FEV1: forced expiratory volume in 1 second, FVC: forced vital capacity, COPD: chronic obstructive pulmonary disease, LLN: lower limit of normal

Table S2: Lung allograft survival analysis by univariate and multivariable time-dependent cox regression analysis, including BLAD grading by FEV1 z-score.

			Univariable Cox Regression Analysis			Multivariable Cox Regression Analysis		
variable	category	n	hazard ratio	95% confidence interval	p-value	hazard ratio	95% confidence interval	p-value
transplant	Bilateral (incl. combined)	624	reference			reference		
	**Unilateral (incl. lobar)**	**102**	**2.349**	**1.703-3.240**	**<0.001**	**0.973**	**0.664-1.425**	**0.887**
BLAD*1	Baseline FEV1 and FVC ≥ LLN	*2	reference			reference		
	**Bsl. FEV1 or FVC < LLN, Z(FEV1) > -2.5**	*2	**1.717**	**1.289-2.287**	**<0.001**	**1.718**	**1.270-2.324**	**<0.001**
	**Bsl. FEV1 or FVC < LLN, Z(FEV1) -2.5 to -4**	*2	**2.131**	**1.471-3.088**	**<0.001**	**2.143**	**1.399-3.281**	**<0.001**
	**Bsl. FEV1 or FVC < LLN, Z(FEV1) < -4**	*2	**12.778**	**1.740-93.857**	**<0.001**	**18.044**	**2.403-135.502**	**0.005**
Obstruction*1	FEV1/FVC ≥ LLN	*2	reference			reference		
	FEV1/FVC < LLN	*2	**1.916**	**1.479-2.483**	**<0.001**	**1.458**	**1.071-1.986**	**0.017**
Diagnosis	COPD/emphysema	201	reference			reference		
	Pulmonary vascular diseases	39	0.931	0.518-1.674	0.810	1.414	0.720-2.775	0.314
	**Cystic fibrosis / bronchiectasis**	**132**	**0.483**	**0.312-0.747**	**<0.001**	0.858	0.484-1.520	0.600
	Pulmonary fibrosis	306	0.859	0.641-1.149	0.306	0.918	0.675-1.249	0.587
	other	48	0.621	0.338-1.139	0.124	0.669	0.354-1.262	0.215
Age	38 - 50 years	155	reference			reference		
	< 38 years	144	1.252	0.795-2.091	0.352	1.198	0.685-2.097	0.527
	**51 - 57 years**	**165**	**1.681**	**1.072-2.592**	**0.018**	**1.847**	**1.182-2.884**	**0.007**
	**58 – 61 years**	**115**	**1.935**	**1.226-3.054**	**0.005**	**2.064**	**1.285-3.315**	**0.003**
	**> 61 years**	**147**	**2.950**	**1.949-4.454**	**<0.001**	**2.999**	**1.895-4.747**	**<0.001**
CLAD*1	No CLAD	*2	reference			reference		
	**BOS phenotype**	*2	**3.791**	**2.697-5.328**	**<0.001**	**3.013**	**2.059-4.407**	**<0.001**
	**RAS/ mixed phenotype**	*2	**5.000**	**3.379-7.400**	**<0.001**	**4.146**	**2.749-6.252**	**<0.001**
	**phenotype, other/ unknown**	*2	**3.941**	**1.595-9.734**	**0.003**	**2.894**	**1.132-7.398**	**0.026**

*1 modelled as time-dependent covariate, *2 modelled as time-dependent covariate; category membership may change over time, hence baseline absolute counts (n) are not applicable.

Footnotes abbreviations: BLAD: baseline lung allograft dysfunction, CLAD: chronic lung allograft dysfunction, BOS: bronchiolitis obliterans syndrome, RAS: restrictive allograft syndrome, FEV1: forced expiratory volume in 1 second, FVC: forced vital capacity, COPD: chronic obstructive pulmonary disease, LLN: lower limit of normal, Bsl: Baseline, Z(FEV1): z-score for FEV1 by GLI reference equation
